# Effect of one‐session focused attention meditation on the working memory capacity of meditation novices: A functional near‐infrared spectroscopy study

**DOI:** 10.1002/brb3.2288

**Published:** 2021-08-03

**Authors:** Noriki Yamaya, Kenji Tsuchiya, Ibuki Takizawa, Kaori Shimoda, Kazuki Kitazawa, Fusae Tozato

**Affiliations:** ^1^ School of Health Sciences, Faculty of Medicine Gunma University Maebashi Japan; ^2^ Department of Rehabilitation Sciences Gunma University Graduate School of Health Sciences Maebashi Japan; ^3^ Department of Occupational Therapy Umayabashi Hospital Maebashi Japan; ^4^ Department of Occupational Therapy Geriatrics Institute and Hospital Maebashi Japan; ^5^ Department of Health Sciences Nagano University of Health and Medicine Nagano Japan

**Keywords:** dorsolateral prefrontal cortex, focused attention meditation, near‐infrared spectroscopy, working memory capacity

## Abstract

**Introduction:**

Previous studies have revealed that one‐session focused attention meditation (FAM) can improve top‐down attention control, which is one of the factors of working memory capacity (WMC). In addition, FAM shares various neural substrates, including the dorsolateral prefrontal cortex (DLPFC), with WMC. Thus, we hypothesized that one‐session FAM would improve WMC by activating the DLPFC evoked by the top‐down attention control. In this study, we examined whether FAM modified WMC in individuals with little to no meditation experience.

**Methods:**

The participants were randomly assigned to either the FAM group (*N* = 13) or the control group (*N* = 17) who engaged in random thinking (i.e., mind‐wandering). Before and after each 15‐min intervention, the participants’ WMC was measured according to the total number of correct answers in the Reading Span Test. During each intervention, functional near‐infrared spectroscopy was employed to measure the blood flow in the participants’ DLPFC and determine the top‐down attention control effect.

**Results:**

In the FAM group, WMC increased, and the bilateral DLPFC was activated during the intervention. As for the control group, WMC decreased after the intervention, and the bilateral DLPFC was not activated during the intervention. A correlation was also found among all participants between the increase in WMC and the activation of the bilateral DLPFC.

**Conclusion:**

The study findings suggest that top‐down attention control during FAM can activate the bilateral DLPFC and increase WMC among meditation novices.

## Graphical abstract

Top‐down attentional control during focused attention meditation can activate the bilateral DLPFC and increase working memory capacity among meditation novices.

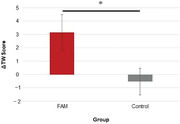

## 1 INTRODUCTION

Cognitive training is an intervention that enhances cognitive functions and improves performance in any daily task underpinned by the trained cognitive functions (Simons et al., 2016; Tang & Posner, 2014), and mindfulness meditation is a type of cognitive training that enhances attentional control, emotional regulation, and self‐awareness by developing brain networks (Tang & Posner, 2014; Tang et al., 2015). Mindfulness meditation can be divided into two main styles: focused attention meditation (FAM) and open monitoring meditation (OMM) (Lutz et al., 2008; Miyoshi et al., 2020). In particular, FAM increases the top‐down attention control as an inhibitory control of attention toward irrelevant stimuli embedded in a cognitive task (Colzato et al., 2015; Colzato et al., 2016; Deepeshwar et al., 2015; Duncan et al., 1997; Lippelt et al., 2014; Wenk‐Sormaz, 2005), whereas OMM decreases the top‐down attention control by encouraging practitioners to spread their attention over an environment (Colzato et al., 2015, 2016). Recently, one‐session FAM was suggested for workplaces (Hafenbrack, 2017) because the benefits of top‐down attention control may underpin certain abilities that influence job performance, such as avoiding distractions at work or concentrating on the task at hand (Fisher et al., 2017). Moreover, as one‐session FAM costs less time and money than long‐term FAM intervention (Hafenbrack, 2017), it will most likely be introduced in other environments such as schools (Waters et al., 2014). Thus, it is necessary to explore and accumulate evidence regarding the effect of one‐session FAM on meditation novices.

FAM is characterized by the dorsolateral prefrontal cortex (DLPFC) activation because FAM entails the top‐down attention control. During FAM, practitioners direct and sustain their attention on a selected object (e.g., breathing or a candle flame) while inhibiting self‐generated thoughts (i.e., mind‐wandering) (Andrews‐Hanna et al., 2014; Lippelt et al., 2014; Lutz et al., 2008). When mind‐wandering occurs, practitioners attempt to detach their attention from it and shift their attention back toward the selected object (Lutz et al., 2008). This top‐down attention control during FAM activates DLPFC, which plays a vital role in the top‐down attention control for visual information processes and behavioral actions (Wang et al., 2020). The DLPFC is believed to be one of the neural substrates of FAM (Fox et al., 2016; Hasenkamp et al., 2012).

As working memory capacity (WMC) supports various daily life functions, such as learning, comprehension, planning, reasoning, and problem‐solving (Cowan, 2014), it has become one of the goals of cognitive training (Constantinidis & Klingberg, 2016). WMC has been characterized as a distributed network in the sensory, parietal, and prefrontal cortices (Christophel et al., 2017). In particular, the DLPFC also plays an essential role in WMC (Barbey et al., 2013; Callicott et al., 1999). Thus, activating the DLPFC can improve WMC (Berryhill & Jones, 2012; Mulquiney et al., 2011; Ohn et al., 2008).

At this point, the following question is raised: What is the effect of one‐session FAM on WMC? One possible answer is that individual differences in WMC are reflected in inhibitory control (Conway & Engle, 1994; Miyake et al., 2000; Osaka, 2006; Tsuchida et al., 2008), and if one‐session FAM can increase such control, it might improve WMC. Another possible answer is that FAM shares the DLPFC with WMC (Christophel et al., 2017; Edin et al., 2009; Gazzaley et al., 2005; Klingberg, 2010; Sreenivasan et al., 2014). Therefore, this study hypothesized that one‐session FAM could improve WMC by activating the DLPFC evoked by the top‐down attention control. To the best of the authors’ knowledge, no study to date has examined the abovementioned aspect.

Therefore, this study compares the effect of one‐session FAM and that of a control intervention method on WMC. For the FAM intervention, Su‐soku meditation (a traditional mindfulness meditation method) was selected (Chiesa, 2009; Lutz et al., 2008). In this method, practitioners must focus on their breathing, count their breathing cycles, and maintain their concentration on their breath to induce the top‐down attention control (Chiesa, 2009; Dunn et al., 1999; Menezes et al., 2013; Park & Park, 2012). As this method does not require any specialized training (Hanh, 2016; Kubota et al., 2001), it is suitable for meditation‐naive participants (Chiesa & Malinowski, 2011; Hanh, 2016). As for the control intervention method, a random‐thinking intervention was selected. This method induces a mental state that differs from that in FAM (Deepeshwar et al., 2015; Telles et al., 2015). In this case, practitioners allow their thoughts to run free, as in mind‐wandering. As mind‐wandering accounts for approximately 47% of everyday thoughts (Killingsworth & Gilbert, 2010), this method is an appropriate control task (Arch & Craske, 2006; Deepeshwar et al., 2015). Moreover, to confirm the influence of the top‐down attention control during the intervention, DLPFC activation was monitored using functional near‐infrared spectroscopy (fNIRS), which includes a high temporal resolution (Hori & Seiyama, 2014) and assesses DLPFC activation during an individual's natural meditation posture. This study thus provides insights into the effect of one‐session FAM on meditation novices.

## 2 MATERIALS AND METHODS

### 2.1 Subjects

In all, 44 healthy university students (seven males and 37 females; mean age = 21.2 ± 1.9 years) with no Su‐soku meditation experience participated in this study after providing their written informed consent. The study protocol was approved by the Gunma University Institutional Review Board (No. 2016–064), and it was registered with the University Hospital Medical Information Network (UMIN) (ID: UMIN000026012).

### 2.2 Experimental procedure

**FIGURE 1:**
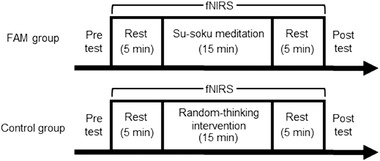
Schematic illustration of the study design. The participants were assigned to either one of the following groups: Focused attention meditation (FAM) or Control groups. Working memory capacity was assessed during the pretest and posttest by using a Reading Span Test. The intervention time was 15 min, and a 5‐min rest period was set before and after each intervention, as a baseline for evaluating the cerebral blood flow concentration of the dorsolateral prefrontal cortex through functional near‐infrared spectroscopy (fNIRS)

Based on a randomized block design, the participants were divided into two groups: FAM and control groups. Then, the participants’ WMC was assessed before and after each 15‐min intervention by using the Japanese (Osaka, 2002) and Gunma University (GU) version (Tsuchiya et al., 2017) of a Reading Span Test (RST) (Daneman & Carpenter, 1980). In this case, the Japanese RST and the GU RST were assigned in a counterbalanced order within each FAM and control group before and after each intervention. In addition, to investigate DLPFC activation by fNIRS, this study employed a single block design, that is, a 5‐min rest period, a 15‐min intervention, and a 5‐min rest period (see Figure 1).

### 2.3 Interventions

First, the participants were asked if they had previous experience in FAM. Then, they were instructed how to conduct each intervention with the help of a paper‐based manual developed by authors after the pretest. They were asked to wear the fNIRS system (explained below) and sit with their jaws fixed on a chin rest. Next, they were instructed to perform the 15‐min FAM or the control intervention with their eyes closed and without any guidance. During the 5‐min rest period, the participants were asked to quietly sit on a chair with their eyes closed. A bell signaled the start and end of each intervention. The participants did not practice each intervention before the experiment. They conducted each intervention in a dim, sound‐proof room to mitigate any noise or light that could have influenced the fNIRS system that they wore.

### 2.4 Su‐soku meditation: FAM group

This study adopted Su‐soku meditation, as described by previous studies (Komuro, 2016; Ryuichiro & Shinobu, 2010). It was conducted for 15 min. Overall, the participants were instructed to perform the following: (1) count their breathing inspiration plus exhalation as one set (i.e., breathing in and out equals one set); (2) count from one to 10 repetitively as they breathe; (3) close their eyes and mentally count their breath; (4) count their breathing sets, despite various thoughts coming to their mind; and (5) in the following instances, recount their breathing from one, and push the counter held in their right hand with their right thumb (Hasenkamp et al., 2012): (a) counting the same number repeatedly; (b) counting by skipping a number; (c) forgetting to count a set; and (d) not counting the number correctly according to their breathing.

### 2.5 Random‐thinking intervention: Control group

The random‐thinking intervention was based on a previous study (Deepeshwar et al., 2015). This control intervention was also conducted for 15 min, which was the same duration as the Su‐soku meditation. Overall, the participants were instructed to listen to randomly recorded conversations or local radio advertisements via speakers to let various thoughts come to their minds. This intervention encourages random thinking because the recorded contents are not connected (Deepeshwar et al., 2015). Moreover, two researchers directly evaluated the participants’ performance and documented their performance on a videotape recorder (HDR‐CX270V; SONY, Corp., Tokyo, Japan) so that the other researchers could check the unrelated tasks of the participants during the intervention, such as physical movements and sleeping.

### 2.6 WMC measurement

The participants’ WMC was evaluated using the Japanese RST and the GU RST (Osaka, 2002; Tsuchiya et al., 2017; Yasumura et al., 2014). In this measurement, the participants were asked to read aloud a sentence and memorize one target word in the sentence. After the participants read several sentences, they were asked to recall the target word included in each sentence. These sentences were presented according to four conditions: two, three, four, and five sentences. Each condition included five trials, and the number of presented sentences increased as the condition numbers increased. For example, a trial in the three‐sentence condition asked the participants to read three sentences aloud and then correctly recall three target words in each sentence. Overall, the participants were shown a total of 70 sentences and 70 target words, including 10 sentences in the two‐sentence condition, 15 in the three‐sentence condition, 20 in the four‐sentence condition, and 25 in the five‐sentence condition.

A previous study reported that one RST high‐score group mainly used a word image strategy in which they created a mental image of the words that they were asked to memorize (Endo & Osaka, 2012). Thus, in the present study, the participants were asked to use the same strategy to memorize the target words. Furthermore, a laptop was employed to present the stimulus sentences. The distance between the laptop and the participant was set at approximately 45 cm. When the trial began, a fixation point was displayed on the screen for 15 s, after which several sentences were presented one‐by‐one according to the condition. Then, a white screen was provided for 5 s per target word in each condition. At that time, the participants were asked to recall the target words that they had memorized. Notably, as the participants were not allowed to report the target word of the final sentence in each trial, a recency effect did not occur.

The RST adopted the following four scoring methods (Friedman & Miyake, 2005). The first method was the “total words (TW)” score, which was the total number of words recalled across trials. The second method was the “proportion words” score, which was the average proportion of words recalled for each trial. The third method was the “correct sets words” score, which was the total number of sentence sets perfectly recalled in a trial based on the conditions. The fourth method was the “span” score, which was the highest‐level conditions score wherein the participants could recall more than three of the five trials correctly based on the conditions (for more details, see Friedman & Miyake, 2005). Among these scoring methods, because of good reliability and normal distributions (Friedman & Miyake, 2005), the TW score was the recommended scoring method for WMC (Robert et al., 2009), even with the various language versions of the RST, such as the English (Friedman & Miyake, 2005), the Japanese (Otsuka & Miyatani, 2007), and other language versions (Schelstraete & Hupet, 2002; Van Den Noort et al., 2008). Previous reports also employed the TW score method to examine the encoding strategies in the RST (Kaakinen & Hyönä, 2007), the relationship between WMC and foreign language proficiency (Van Den Noort et al., 2006), the age difference in WMC (Robert et al., 2009), and the relationship between WMC and reading comprehension ability (Friedman & Miyake, 2004). Thus, we selected the TW score to evaluate WMC (maximum = 70 words).

### 2.7 fNIRS recording

A 42‐channel fNIRS system (LABNIRS; Shimadzu, Corp., Kyoto, Japan) was used to measure the cerebral blood flow concentration. The probe arrangement was fitted to cover the prefrontal cortex, according to the international 10−20 method Fpz. The probes were placed in vertical(*n* = 3) and horizontal (*n* = 9) at 3‐cm intervals, with two probes placed on the left and the right to measure the skin blood flow concentration. The distance between these two probes was set at 1.5 cm. In addition, the researchers measured the fNIRS optical density data in oxygenated hemoglobin (oxy‐Hb), deoxygenated hemoglobin, and total hemoglobin and converted them into a concentration change every 0.1 s, according to the modified Beer–Lambert law (Delpy et al., 1988). After the measurement, the positional information for each probe was obtained by using a three‐dimensional digitizer (FASTRAK; Polhemus, Colchester, VT, USA). In this case, the reference points included the nasal root (Nz), the parietal (Cz), and the left and right preauricular points.

To remove some noise signals, we preprocessed the concentration changes in the following manner. First, a segment‐independent component analysis was conducted to remove the skin blood flow rate. This method was installed as an analysis toolbox on the fNIRS system. The researchers calculated the spatial uniformity coefficient to evaluate the skin blood flow rate in these concentration changes. Then, the estimated skin blood flow rate was removed from the raw data through the independent component analysis (Kohno et al., 2007). Second, the researchers used 2.5‐s moving average processing to smooth out these concentration changes, which entailed removing high‐frequency components due to bodily movements and heartbeats (Koo et al., 2015; Nishimura et al., 2009). After preprocessing, the concentration changes were summed up every 5 min to extract the integrated values, that is, two integrated values during the rest period and three integrated values during the intervention.

Overall, the purpose was to detect any hemodynamic responses on both sides of the DLPFC, which were established as the regions of interest (ROIs). These ROIs were identified by using statistical parametric mapping for fNIRS from the obtained positional information of each probe (Ye et al., 2009). The left and right DLPFCs were set as each channel by using probabilistic registration (Singh et al., 2005). More specifically, the Montreal Neurological Institute coordinates (Brett et al., 2002) were calculated from the positional information obtained from the three‐dimensional digitizer. Then, the computed channels (based on Brodmann areas) were labeled according to the Talairach Daemon atlas (Lancaster et al., 2000), an international anatomical labeling application.

**FIGURE 2:**
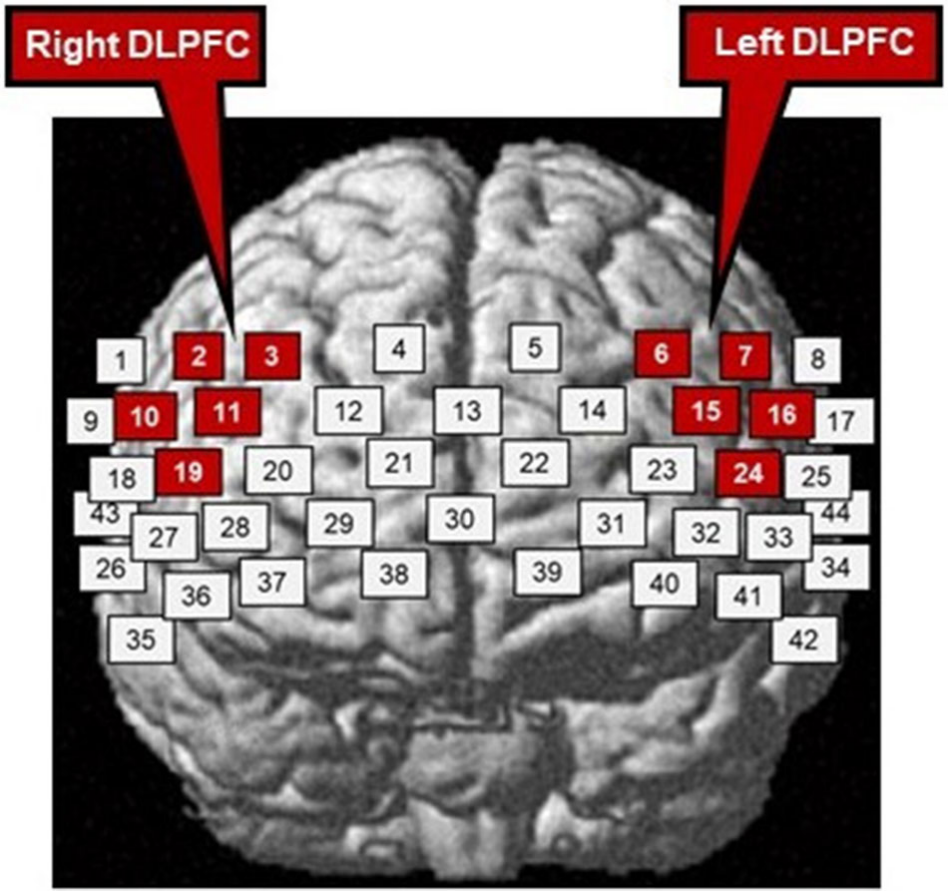
Positions of the functional near‐infrared spectroscopy (fNIRS) channels, with white and red squares indicating the fNIRS channel positions. Only red clusters indicate the region of interest, that is, the dorsolateral prefrontal cortex (DLPFC)

Furthermore, this study used five adjacent channels corresponding to the two ROIs, that is, right DLPFC (Channels 2, 3, 10, 11, and 19) and left DLPFC (Channels 6, 7, 15, 16, and 24) (see Figure 2). This procedure was considered reasonable because the adjacent channels included similar light components (Katagiri et al., 2010). Moreover, as oxy‐Hb is the most sensitive index regarding changes in fNIRS measurement values, the oxy‐Hb data were reported as the primary fNIRS outcome (Hoshi et al., 2001).

## 3 DATA ANALYSIS

Overall, the data from 14 participants were excluded from the analysis because they did not meet the experimental requirements. In this regard, four lost data because of technical issues, four failed to distinguish FAM from the rest period, three fell asleep, and three frequently moved their hands, arms, torsos, and legs during the intervention. Consequently, the analysis included the data from the remaining 30 participants (four males and 26 females; mean age = 21.1 ± 1.8 years; FAM group = 13, control group = 17). Some of the participants (*N* = 6) had previous experience in other types of meditations. However, none of the participants had meditated for 5 min or longer per day within the past month, which was a criterion for a novice meditator (Atchley et al., 2016).

### 3.1 RST measurement analysis

As the study's purpose is to assess the effect of FAM on WMC, the researchers calculated the change in the TW score (i.e., the posttest score minus the pretest score: ΔTW). Next, an analysis of covariance (ANCOVA) was conducted for the ΔTW score, with the ΔTW score as the dependent variable and the type of group (i.e., FAM or control) as the independent variable. Additionally, the pretest TW score was the covariate, according to previous studies (Nouchi, Saito, et al., 2016; Nouchi et al., 2013; Nouchi, Taki, et al., 2016). Previous research indicates that an ANCOVA can reduce error variance (Takeuchi et al., 2016) and control the effects of pre‐intervention values (Nouchi, Saito, et al., 2016; Nouchi et al., 2013; Nouchi, Taki, et al., 2016). Moreover, the researchers calculated the effect size, that is, partial eta squared (*η*_p_^2^) (small = .01; medium = .06; large = .14) (Cohen, 1988).

### 3.2 fNIRS analysis

To assess the effect of FAM on brain activity, we averaged two integrated values of rest periods and three integrated values of intervention times for each. Then, the change in the oxy‐Hb value for the right and left ROI of each group was calculated (i.e., intervention time minus rest period: Δoxy‐Hb). To compare the Δoxy‐Hb between groups, we converted the Δoxy‐Hb of the ROIs into *Z*‐scores by using the following formula: *Z* = (*X* − *μ*) / σ, where the mean value is 0, and the standard deviation is 1 (Tsunashima & Yanagisawa, 2009). In this formula, *X* is the Δoxy‐Hb of the ROI, *μ* is their mean value, and *σ* is the standard deviation. A two‐way repeated measures analysis of variance (RM‐ANOVA) was employed to evaluate the differences in Δoxy‐Hb between the ROIs (i.e., right or left) and the groups (i.e., FAM or control). In this case, if an ROI by the group interaction effect was confirmed, then a post hoc analysis with Shaffer's procedure for multiple comparisons was conducted between the mean values of the different ROIs and groups.

Finally, to examine the relationship between the ΔTW score and the Δoxy‐Hb, Pearson's correlation coefficient was calculated between the ΔTW score and the Δoxy‐Hb. If an ROI by the group interaction effect was confirmed, then the Pearson correlation coefficient was calculated for each ROI. If not, then the right and left Δoxy‐Hb (bilateral Δoxy‐Hb) were averaged in each group, and the Pearson correlation coefficient was calculated. Moreover, the researchers calculated the effect size, that is, partial eta squared (*η*_p_^2^) (small = .01; medium = .06; large = .14) (Cohen, 1988), and the data were analyzed by using R version 4.0.2 software, with a significance level of *p *< .05.

## 4 RESULTS

### 4.1 RST measure

**FIGURE 3:**
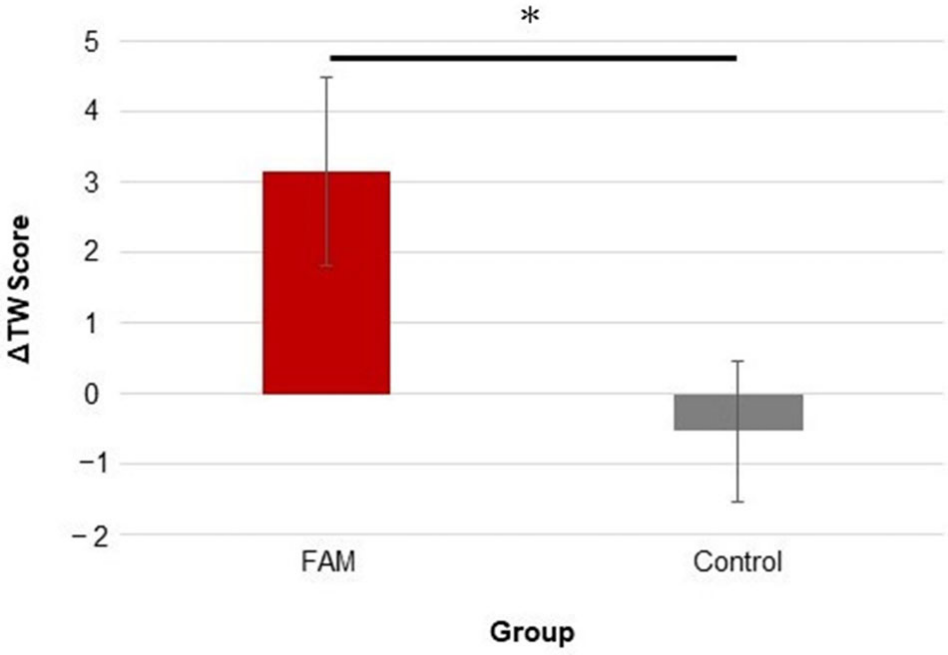
Change in the total word score (i.e., the posttest score minus the pretest score: ΔTW) for each focused attention meditation (FAM) group and control intervention group. The error bar represents the standard error of the mean. *p < .05

Overall, the ΔTW score for the FAM group was 3.2 ± 1.3, whereas the ΔTW score for the control group was −0.53 ± 1.0. Additionally, the results of the ANCOVA revealed a significant difference between the groups (*F* [1, 27] = 6.89, *η*_p_^2^ = .20, 95% family‐wise CI [0.96, 7.8], *p* = .014; see Figure 3). This finding suggests that FAM improved the WMC of the participants, as opposed to the control group.

### 4.2 fNIRS data

**FIGURE 4:**
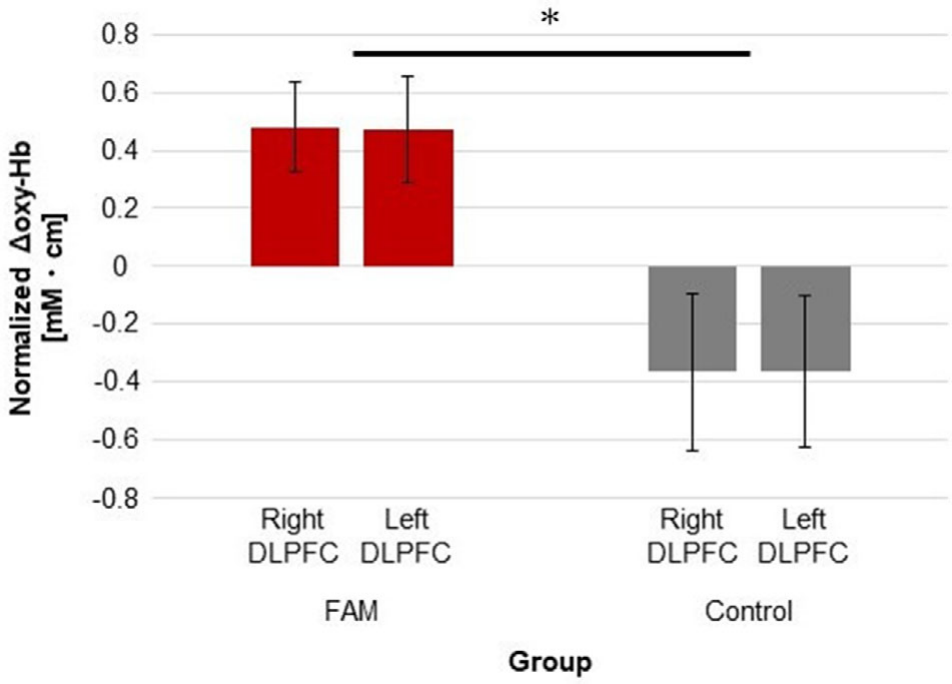
Normalized change value in the integrated value of oxy‐Hb (i.e., the intervention time minus the rest period: Δoxy‐Hb) for the right and left dorsolateral prefrontal cortex (DLPFC) of each focused attention meditation (FAM) group and control intervention group. The error bar represents the standard error of the mean. *p < .05

According to the results of the RM‐ANOVA for the ROIs (i.e., the right and left) and the groups (i.e., FAM and control), no significant effect was observed for the ROIs; however, a significant effect was observed for the groups (Greenhouse–Geisser epsilon correction: *F* [1, 28] = 6.66, *η*_p_^2^ = .19, *p* = .015). In addition, no significant interaction was found for ROIs × groups (Greenhouse–Geisser epsilon correction: *F* [1, 28] = 2.50 × 10^–3^, *η*_p_^2^ = .10 × 10^–3^, *p* = .96; see Table 1 and Figure 4). These findings suggest that FAM increased oxy‐Hb in the right and left DLPFCs more than the control intervention method.

**TABLE 1:** Δoxy‐Hb signals in the regions of interest (ROIs) in the focused attention meditation (FAM) group and the random‐thinking intervention (control) group

Group	*n*	ROI	Mean	SEM	CI (95%)	
		(DLPFC)			Minimum	Maximum
FAM	13	Right	0.48	0.16	0.14	0.82
		Left	0.47	0.18	0.13	0.81
Control	17	Right	−0.37	0.27	−0.71	−0.025
		Left	−0.36	0.26	−0.70	−0.021

*Note*: This study calculated Loftus–Masson's Difference‐Adjusted Pooled Confidence Intervals (CIs). Abbreviations: DLPFC, dorsolateral prefrontal cortex; SEM, standard error of the mean.

**FIGURE 5:**
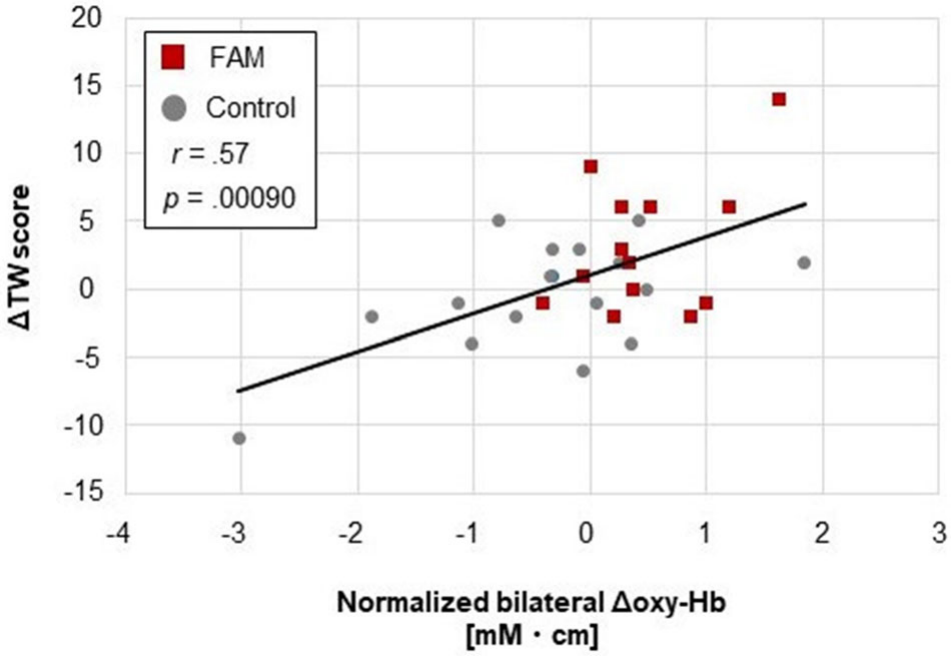
Relationship between the change in the total word score (i.e., the posttest score minus the pretest score: ΔTW) and the normalized change value in the integrated value of oxy‐Hb (i.e., the intervention time minus the rest period: Δoxy‐Hb) for the bilateral dorsolateral prefrontal cortex (DLPFC) of each focused attention meditation (FAM) group and control intervention group

Finally, Figure 5 presents the correlation between the ΔTW scores and the bilateral Δoxy‐Hb for all of the study participants. In this case, a significant positive correlation was found between the ΔTW scores and the bilateral Δoxy‐Hb (*r *= .57, *p* = .90 × 10^–3^). This finding suggests that the increase in bilateral DLPFC activation was related to WMC improvement.

## 5 DISCUSSION

This study investigated the effect of one‐session FAM on WMC compared to the random‐thinking intervention as the control intervention method. It also used fNIRS to assess the hemodynamic DLPFC responses in both interventions and support the top‐down attention control effect. This report was the first to examine the effect of one‐session FAM on WMC among meditation novices and its mechanism from a neuroscientific perspective. The results demonstrated that after the meditation, the participants in the FAM group possessed an average of three more words among the total number of words that they had memorized across the trials than before the meditation. In contrast, the participants in the control group slightly decreased the total number of words that they had memorized across the trials. As expected, the change in the TW score (from before to after the intervention) was significantly larger in the FAM group than in the control group, with a large effect size of the difference (*η*_p_^2^ = .20) (Cohen, 1988). This result suggested that FAM improved WMC more than the control intervention method (see Figure 3). Additionally, there are two perspectives for interpreting the findings: (1) the effect of the top‐down attention control and (2) the effect of change in consciousness caused by the top‐down attention control (Hölzel et al., 2011).

The top‐down attention control is one of the important components for producing the efficacy of mindfulness meditation (Hölzel et al., 2011). As for the Su‐soku meditation in the FAM group, the participants were asked to focus on their breathing while counting their respiratory rate. If mind‐wandering occurred, then they had to shift their attention back toward their breathing. This top‐down attention control includes an inhibitory control to mind‐wandering (Malinowski, 2013; Markowska, 2013). Previous studies have also reported that one‐session FAM improved inhibitory control (Deepeshwar et al., 2015; Wenk‐Sormaz, 2005). In the current study, WMC was evaluated by RST, which asked the participants to read aloud some sentences, including irrelevant words and specific keywords, and memorize the specific keywords. Thus, their RST performance was influenced by inhibitory control of attention toward irrelevant information (Conway & Engle, 1994; Miyake et al., 2000; Osaka, 2006; Tsuchida et al., 2008). Based on these findings, it is possible to conclude that repeated top‐down attention control during FAM enhances inhibitory control toward irrelevant information, such as unrelated words (Osaka, 2006; Osaka et al., 2002), and leads to improved RST.

The results of the fNIRS also support this suggestion. This study confirmed that FAM increased bilateral DLPFC activation more than the control group (see Figure 4). It also found that the increase in bilateral DLPFC activation was related to WMC improvement (see Figure 5). Similarly, previous studies show that the DLPFC is related to the top‐down attention control during FAM (Hasenkamp et al., 2012; Lippelt et al., 2014), a critical role in inhibitory control (Aron et al., 2014; Cipolotti et al., 2016; Dias et al., 1997; Lau et al., 2006; Ridderinkhof et al., 2004), and in RST performance (publisher). Thus, this study suggested that inhibitory control induced by the top‐down attention control in FAM evokes DLPFC activation, which, in turn, leads to WMC improvement (Curtis & D'Esposito, 2003; Lau et al., 2006; Ridderinkhof et al., 2004; Seeley et al., 2007; Taren et al., 2017).

Contrary to FAM, the control intervention method required less top‐down attention control. The participants in the control group thought freely about various topics and ideas, and it was stimulated by listening to short dialogues, advertisements, and other inputs. As this mental process includes no specific purpose, it is similar to mind‐wandering (Deepeshwar et al., 2015; Smallwood & Schooler, 2006). The fNIRS results showing the deactivation of the bilateral DLPFC in the control intervention also supported the induced mind‐wandering. Previous studies show that mind‐wandering is related to default network activation (Andrews‐Hanna et al., 2014; Christoff et al., 2009; Mason et al., 2007). This network is mainly involved in the medial prefrontal cortex and posterior cingulate gyrus (Raichle et al., 2001). Based on these findings, the control intervention method in this study did not activate the DLPFC. Moreover, a previous study reported that during mind‐wandering, one's ability to perform WMC tasks is often attenuated (Smallwood & Schooler, 2006). Thus, this study suggests that mind‐wandering in the control group did not improve RST performance.

In addition to the aforementioned effect of the top‐down attention control, participants’ consciousness could have affected WMC (Hölzel et al., 2011). According to the Sphere Model of Consciousness (Paoletti & Ben‐Soussan, 2019), the top‐down attention control induced by meditation changes participants’ consciousness from the *narrative self* to the *minimal self* and further to *overcoming of the self* (Paoletti & Ben‐Soussan, 2019, 2020; Pintimalli et al., 2020). In this respect, the *narrative self* is a consciousness that comprises various stories, including the past and future representation with emotional valence (Gallagher, 2000). It corresponds to a mind‐wandering state supported by the default network or the hippocampal–cortical memory system (Andrews‐Hanna et al., 2014; Christoff et al., 2009; Mason et al., 2007; Paoletti & Ben‐Soussan, 2019; Vago & Silbersweig, 2012; Vincent et al., 2008). As for the *minimal self*, it is a consciousness made by an immediate subject of experience in the present (Gallagher, 2000). The *minimal self* also refers to higher‐order conscious or volitional awareness supported by the dorsal attention and frontoparietal networks, including the DLPFC, dorsal anterior cingulate cortex, and temporoparietal junction (Paoletti & Ben‐Soussan, 2019; Vago & Silbersweig, 2012). Finally, the *overcoming of the self* refers to a deep state of silence or consciousness without contents supported by the multiple demand system (Duncan, 2010; Raffone & Srinivasan, 2017), particularly the insula cortex (Paoletti & Ben‐Soussan, 2020). Additionally, the changed consciousness during meditation affects the state of consciousness after meditation. Previous studies indicate that the *minimal self* and the *overcoming of the self*, induced by meditation, can lead to increased meta‐awareness (Vago & Silbersweig, 2012) and a change in consciousness after meditation (Pintimalli et al., 2020). In this study, we found significant DLPFC activations evoked by FAM, which suggested that the *minimal self* or the *overcoming of the self* was induced during FAM and continued after FAM. Thus, the increased WMC can be explained by the effects of the top‐down attention control and the effect of change in consciousness caused by the top‐down attention control.

In contrast to previous findings on the effect of one‐session OMM (Banks et al., 2015), this study found that one‐session meditation improved WMC. Regarding the differences between these two methods, OMM does not focus on a particular object (e.g., breathing and a candle flame) but allows practitioners to notice any thoughts or experiences that might arise during meditation (Lutz et al., 2008). Previous studies have reported that OMM encourages the spreading of one's attention over an environment and decreases the top‐down attention control (Colzato et al., 2015, 2016). In this regard, as different meditation methods have different effects on cognitive domains (Lippelt et al., 2014), it is essential to verify the effectiveness of each meditation type. For example, some previous studies have independently examined the effect of one‐session FAM or one‐session OMM on memory function (Eisenbeck et al., 2018) and executive function (Luu & Hall, 2017; Wenk‐Sormaz, 2005). Thus, it can be useful to compare one‐session FAM and one‐session OMM among novice meditators to clarify the effectiveness of meditation type depending on the cognitive domain.

Finally, this study has five noteworthy limitations. First, although the change in consciousness could be explained by the DLPFC activation (Vago & Silbersweig, 2012), it may be better to confirm the change in consciousness by adding an assessment of the subjective experience such as the Tennessee Self‐Concept Scale (Fitts & Roid, 1989; Hölzel et al., 2011). From the standpoint of the characteristics of the *narrative self* and the *minimal self*, it may be useful to assess the frequency of mind‐wandering during the interventions through a Likert scale to distinguish the consciousness between the interventions (Garrison et al., 2014; Garrison et al., 2015). However, further study is necessary to confirm this suggestion. Second, we did not assess the quality of the participants’ FAM. The researchers acknowledge that it would have been better to confirm such quality by performing a subjective assessment, as in previous studies (Garrison et al., 2014, 2015). However, it was assumed that the participants could have effectively implemented FAM in compliance with the presented instruction. In this regard, previous studies have shown that Su‐soku meditation is appropriate for meditation‐naive participants because it does not require specialized training (Chiesa & Malinowski, 2011; Hanh, 2016; Takahashi et al., 2005). Related studies have also shown that novice participants who do not practice daily meditation can perform it effectively in short durations (Miyoshi et al., 2020; Murata et al., 2004; Takahashi et al., 2005). Meanwhile, this study excluded any participants who moved their hands, arms, torsos, and legs during the intervention as well as those who could not distinguish the interventions from the rest periods. Thus, this study's results are based on the notion that the participants could have completed FAM correctly and effectively. Third, it is also important to note one aspect of the interventions that could have affected the results. Specifically, the FAM group contained a motor component related to the counter‐pushing task, whereas the control group did not include this component. However, it was assumed that the motor response did not significantly influence the results. According to a previous study (Hasenkamp et al., 2012), which focused on the participants’ awareness of mind‐wandering by button‐pressing, such actions did not influence DLPFC activation. Similarly, another study showed that an easy rhythmic tapping task did not contribute to DLPFC activation (Abiru et al., 2016). Thus, it is possible to conclude that the FAM group's motor response did not significantly affect the bilateral DLPFC activation and WMC improvement. Fourth, other brain regions may have contributed to WMC improvement. Previous studies show that FAM activates other brain regions, including the anterior cingulate cortex and insula (Fox et al., 2016; Hasenkamp et al., 2012). Considering the aforementioned Sphere Model of Consciousness (Paoletti & Ben‐Soussan, 2019), we can assume that the frontoparietal network, dorsal attention network, and multiple demand system might also be associated with WMC improvement. Future research should investigate the activation of other brain regions during and after one‐session FAM and their contributions toward WMC improvement. Fifth, all of the participants were young adults. As the effect of one‐session FAM on the WMC of older adults and children is still unknown, future studies should focus on these age groups to generalize the study findings.

## 6 CONCLUSION

This study was the first to investigate the effects of one‐session FAM on the WMC of meditation novices and monitor their DLPFC activation using fNIRS to determine its mechanism from a neuroscientific perspective. The study results revealed an increase in WMC after FAM compared to the random‐thinking intervention as the control intervention method. A significant bilateral DLPFC activation was also observed in contrast to the control intervention method, which was correlated with WMC improvement. Overall, we conclude that one‐session FAM can improve WMC through DLPFC activation, stimulated by top‐down attention control (including inhibitory control) or by a change in consciousness evoked by such control. It can be helpful to conduct one‐session FAM before a class at school or before a job at a workplace to deal with difficulties more efficiently. Moreover, these results may contribute to evidence regarding the effect of one‐session FAM on meditation novices.

## CONFLICT OF INTEREST

The authors declare no conflict of interest.

## AUTHOR CONTRIBUTIONS

NY, KT, KS, and FT conceived this study. NY, KT, IT, KS, and FT designed the methodology. NY and IT contributed to the investigation. NY, KT, and KK contributed to the formal analysis. NY helped write the original draft. FT offered the resources and contributed to the supervision and administration of the project. All of the authors have read and approved the final manuscript.

## PEER REVIEW

The peer review history for this article is available at https://publons.com/publon/10.1002/brb3.2288

## Data Availability

The data are not publicly available due to ethical restrictions, although permission to share the data was obtained from the study participants. However, the data that support the study findings are available on request from the corresponding author.

## References

[brb32288-bib-0001] Abiru, M., Sakai, H., Sawada, Y., & Yamane, H. (2016). The effect of the challenging two handed rhythm tapping task to DLPFC activation. Asian Journal of Occupational Therapy, 12(1), 75–83. 10.11596/asiajot.12.75

[brb32288-bib-0002] Andrews‐Hanna, J. R., Smallwood, J., & Spreng, R. N. (2014). The default network and self‐generated thought: Component processes, dynamic control, and clinical relevance. Annals of the New York Academy of Sciences, 1316(1), 29–52. 10.1111/nyas.12360 24502540 PMC4039623

[brb32288-bib-0003] Arch, J. J., & Craske, M. G. (2006). Mechanisms of mindfulness: Emotion regulation following a focused breathing induction. Behaviour Research and Therapy, 44(12), 1849–1858. 10.1016/j.brat.2005.12.007 16460668

[brb32288-bib-0004] Aron, A. R., Robbins, T. W., & Poldrack, R. A. (2014). Inhibition and the right inferior frontal cortex: One decade on. Trends in Cognitive Sciences, 18(4), 177–185. 10.1016/j.tics.2013.12.003 24440116

[brb32288-bib-0005] Atchley, R., Klee, D., Memmott, T., Goodrich, E., Wahbeh, H., & Oken, B. (2016). Event‐related potential correlates of mindfulness meditation competence. Neuroscience, 320, 83–92. 10.1016/j.neuroscience.2016.01.051 26850995 PMC4777645

[brb32288-bib-0006] Banks, J. B., Welhaf, M. S., & Srour, A. (2015). The protective effects of brief mindfulness meditation training. Consciousness and Cognition, 33, 277–285. 10.1016/j.concog.2015.01.016 25680006

[brb32288-bib-0007] Barbey, A. K., Koenigs, M., & Grafman, J. (2013). Dorsolateral prefrontal contributions to human working memory. Cortex; a Journal Devoted to the Study of the Nervous System and Behavior, 49(5), 1195–1205. 10.1016/j.cortex.2012.05.022 22789779 PMC3495093

[brb32288-bib-0008] Berryhill, M. E., & Jones, K. T. (2012). tDCS selectively improves working memory in older adults with more education. Neuroscience Letters, 521(2), 148–151. 10.1016/j.neulet.2012.05.074 22684095

[brb32288-bib-0009] Brett, M., Johnsrude, I. S., & Owen, A. M. (2002). The problem of functional localization in the human brain. Nature Reviews Neuroscience, 3(3), 243–249. 11994756 10.1038/nrn756

[brb32288-bib-0010] Callicott, J. H., Mattay, V. S., Bertolino, A., Finn, K., Coppola, R., Frank, J. A., Goldberg, T. E., & Weinberger, D. R. (1999). Physiological characteristics of capacity constraints in working memory as revealed by functional MRI. Cerebral Cortex, 9(1), 20–26. 10.1093/cercor/9.1.20 10022492

[brb32288-bib-0011] Chiesa, A. (2009). Zen meditation: An integration of current evidence. Journal of Alternative and Complementary Medicine, 15(5), 585–592. 10.1089/acm.2008.0416 19422285

[brb32288-bib-0012] Chiesa, A., & Malinowski, P. (2011). Mindfulness‐based approaches: Are they all the same? Journal of Clinical Psychology, 67(4), 404–424. 10.1002/jclp.20776 21254062

[brb32288-bib-0013] Christoff, K., Gordon, A. M., Smallwood, J., Smith, R., & Schooler, J. W. (2009). Experience sampling during fMRI reveals default network and executive system contributions to mind wandering. Proceedings of the National Academy of Sciences of the United States of America, 106(21), 8719–8724. 10.1073/pnas.0900234106 19433790 PMC2689035

[brb32288-bib-0014] Christophel, T. B., Klink, P. C., Spitzer, B., Roelfsema, P. R., & Haynes, J. D. (2017). The distributed nature of working memory. Trends in Cognitive Sciences, 21(2), 111–124. 10.1016/j.tics.2016.12.007 28063661

[brb32288-bib-0015] Cipolotti, L., Spanò, B., Healy, C., Tudor‐Sfetea, C., Chan, E., White, M., Biondo, F., Duncan, J., Shallice, T., & Bozzali, M. (2016). Inhibition processes are dissociable and lateralized in human prefrontal cortex. Neuropsychologia, 93, 1–12. 10.1016/j.neuropsychologia.2016.09.018 27671485

[brb32288-bib-0016] Cohen, J. (1988). Statistical power analysis for the behavioral sciences (2nd ed.). Lawrence Erlbaum Associates. 10.4324/9780203771587

[brb32288-bib-0017] Colzato, L. S., Sellaro, R., Samara, I., Baas, M., & Hommel, B. (2015). Meditation‐induced states predict attentional control over time. Consciousness and Cognition, 37, 57–62. 10.1016/j.concog.2015.08.006 26320866

[brb32288-bib-0018] Colzato, L. S., van der Wel, P., Sellaro, R., & Hommel, B. (2016). A single bout of meditation biases cognitive control but not attentional focusing: Evidence from the global–local task. Consciousness and Cognition, 39, 1–7. 10.1016/j.concog.2015.11.003 26637968

[brb32288-bib-0019] Constantinidis, C., & Klingberg, T. (2016). The neuroscience of working memory capacity and training. Nature Reviews Neuroscience, 17(7), 438–449. 10.1038/nrn.2016.43 27225070

[brb32288-bib-0020] Conway, A. R. A., & Engle, R. W. (1994). Working memory and retrieval: A resource‐dependent inhibition model. Journal of Experimental Psychology: General, 123(4), 354–373. 10.1037/0096-3445.123.4.354 7996121

[brb32288-bib-0021] Cowan, N. (2014). Working memory underpins cognitive development, learning, and education. Educational Psychology Review, 26(2), 197–223. 10.1007/s10648-013-9246-y 25346585 PMC4207727

[brb32288-bib-0022] Curtis, C. E., & D'Esposito, M. (2003). Persistent activity in the prefrontal cortex during working memory. Trends in Cognitive Sciences, 7(9), 415–423. 10.1016/S1364-6613(03)00197-9 12963473

[brb32288-bib-0023] Daneman, M., & Carpenter, P. A. (1980). Individual differences in working memory and reading. Journal of Verbal Learning and Verbal Behavior, 19(4), 450–466. 10.1016/j.gassur.2003.10.009

[brb32288-bib-0024] Deepeshwar, S., Vinchurkar, S. A., Visweswaraiah, N. K., & Nagendra, H. R. (2015). Hemodynamic responses on prefrontal cortex related to meditation and attentional task. Frontiers in Systems Neuroscience, 8, 1–13. 10.3389/fnsys.2014.00252 PMC4330717 25741245

[brb32288-bib-0025] Delpy, D. T., Cope, M., Van Der Zee, P., Arridge, S., Wray, S., & Wyatt, J. (1988). Estimation of optical pathlength through tissue from direct time of flight measurement. Physics in Medicine and Biology, 33(12), 1433–1442. 10.1088/0031-9155/33/12/008 3237772

[brb32288-bib-0026] Dias, R., Robbins, T. W., & Roberts, A. C. (1997). Dissociable forms of inhibitory control within prefrontal cortex with an analog of the Wisconsin card sort test: Restriction to novel situations and independence from “on‐line” processing. Journal of Neuroscience, 17(23), 9285–9297. 9364074 10.1523/JNEUROSCI.17-23-09285.1997 PMC6573594

[brb32288-bib-0027] Duncan, J. (2010). The multiple‐demand (MD) system of the primate brain: Mental programs for intelligent behaviour. Trends in Cognitive Sciences, 14(4), 172–179. 10.1016/j.tics.2010.01.004 20171926

[brb32288-bib-0028] Duncan, J., Humphreys, G., & Ward, R. (1997). Competitive brain activity in visual attention. Current Opinion in Neurobiology, 7(2), 255–261. 10.1016/S0959-4388(97)80014-1 9142748

[brb32288-bib-0029] Dunn, B. R., Hartigan, J. A., & Mikulas, W. L. (1999). Concentration and mindfulness meditations: Unique forms of consciousness? Applied Psychophysiology Biofeedback, 24(3), 147–165. 10.1023/A:1023498629385 10652635

[brb32288-bib-0030] Edin, F., Klingberg, T., Johansson, P., McNab, F., Tegnér, J., & Compte, A. (2009). Mechanism for top‐down control of working memory capacity. Proceedings of the National Academy of Sciences, 106(16), 6802–6807. 10.1073/pnas.0901894106 PMC2672558 19339493

[brb32288-bib-0031] Eisenbeck, N., Luciano, C., & Valdivia‐Salas, S. (2018). Effects of a focused breathing mindfulness exercise on attention, memory, and mood: The importance of task characteristics. Behaviour Change, 35(1), 54–70. 10.1017/bec.2018.9

[brb32288-bib-0032] Endo, K., & Osaka, M. (2012). Nihongoban reading span test ni okeru houryakuriyou no kojinsa [Individual differences in strategy use in the Japanese Reading Span Test]. Japanese Journal of Psychology, 82(6), 554–559. 10.4992/jjpsy.82.554 22514908

[brb32288-bib-0033] Fisher, G. G., Chaffee, D. S., Tetrick, L. E., Davalos, D. B., & Potter, G. G. (2017). Cognitive functioning, aging, and work: A review and recommendations for research and practice. Journal of Occupational Health Psychology, 22(3), 314–336. 10.1037/ocp0000086 28358568

[brb32288-bib-0034] Fitts, W., & Roid, G. H. (1989). Tennessee self concept scale: Revised manual. Western Psychological Services.

[brb32288-bib-0035] Fox, K. C. R., Dixon, M. L., Nijeboer, S., Girn, M., Floman, J. L., Lifshitz, M., Ellamil, M., Sedlmeier, P., & Christoff, K. (2016). Functional neuroanatomy of meditation: A review and meta‐analysis of 78 functional neuroimaging investigations. Neuroscience and Biobehavioral Reviews, 65, 208–228. 10.1016/j.neubiorev.2016.03.021 27032724

[brb32288-bib-0036] Friedman, N. P., & Miyake, A. (2004). The reading span test and its predictive power for reading comprehension ability. Journal of Memory and Language, 51(1), 136–158. 10.1016/j.jml.2004.03.008

[brb32288-bib-0037] Friedman, N. P., & Miyake, A. (2005). Comparison of four scoring methods for the reading span test. Behavior Research Methods, 37(4), 581–590. 10.3758/BF03192728 16629290

[brb32288-bib-0038] Gallagher, S. (2000). Philosophical conceptions of the self: Implications for cognitive science. Trends in Cognitive Sciences, 4(1), 14–21. 10.1016/S1364-6613(99)01417-5 10637618

[brb32288-bib-0039] Garrison, K. A., Scheinost, D., Constable, R. T., & Brewer, J. A. (2014). BOLD signal and functional connectivity associated with loving kindness meditation. Brain and Behavior, 4(3), 337–347. 10.1002/brb3.219 24944863 PMC4055184

[brb32288-bib-0040] Garrison, K. A., Zeffiro, T. A., Scheinost, D., Constable, R. T., & Brewer, J. A. (2015). Meditation leads to reduced default mode network activity beyond an active task. Cognitive, Affective and Behavioral Neuroscience, 15(3), 712–720. 10.3758/s13415-015-0358-3 PMC4529365 25904238

[brb32288-bib-0041] Gazzaley, A., Cooney, J. W., Rissman, J., & D'Esposito, M. (2005). Top‐down suppression deficit underlies working memory impairment in normal aging. Nature Neuroscience, 8(10), 1298–1300. 10.1038/nn1543 16158065

[brb32288-bib-0042] Hafenbrack, A. C. (2017). Mindfulness meditation as an on‐the‐spot workplace intervention. Journal of Business Research, 75, 118–129. 10.1016/j.jbusres.2017.01.017

[brb32288-bib-0043] Hanh, T. N. (2016). The miracle of mindfulness: An introduction to the practice of meditation. Beacon Press.

[brb32288-bib-0044] Hasenkamp, W., Wilson‐Mendenhall, C. D., Duncan, E., & Barsalou, L. W. (2012). Mind wandering and attention during focused meditation: A fine‐grained temporal analysis of fluctuating cognitive states. Neuroimage, 59(1), 750–760. 10.1016/j.neuroimage.2011.07.008 21782031

[brb32288-bib-0045] Hölzel, B. K., Lazar, S. W., Gard, T., Schuman‐Olivier, Z., Vago, D. R., & Ott, U. (2011). How does mindfulness meditation work? Proposing mechanisms of action from a conceptual and neural perspective. Perspectives on Psychological Science, 6(6), 537–559. 10.1177/1745691611419671 26168376

[brb32288-bib-0046] Hori, S., & Seiyama, A. (2014). Regulation of cerebral blood flow during stimulus‐induced brain activation: Instructions for the correct interpretation of fNIRS signals. Journal of Physical Fitness and Sports Medicine, 3(1), 91–100. 10.7600/jpfsm.3.91

[brb32288-bib-0047] Hoshi, Y., Kobayashi, N., & Tamura, M. (2001). Interpretation of near‐infrared spectroscopy signals: A study with a newly developed perfused rat brain model. Journal of Applied Physiology, 90(5), 1657–1662. http://www.ncbi.nlm.nih.gov/pubmed/11299252 11299252 10.1152/jappl.2001.90.5.1657

[brb32288-bib-0048] Kaakinen, J. K., & Hyönä, J. (2007). Strategy use in the reading span test: An analysis of eye movements and reported encoding strategies. Memory, 15(6), 634–646. 10.1080/09658210701457096 17654278

[brb32288-bib-0049] Katagiri, A., Dan, I., Tuzuki, D., Okamoto, M., Yokose, N., Igarashi, K., Hoshino, T., Fujiwara, T., Katayama, Y., Yamaguchi, Y., & Sakatani, K. (2010). Mapping of optical pathlength of human adult head at multi‐wavelengths in near infrared spectroscopy. In E. Takahashi & D. F. Bruley (Eds.), Oxygen transport to tissue XXXI (Vol. 662, pp. 205–212). Springer. 10.1007/978-1-4419-1241-1 20204793

[brb32288-bib-0050] Killingsworth, M. A., & Gilbert, D. T. (2010). A wandering mind is an unhappy mind. Science, 330(6006), 932. 10.1126/science.1192439 21071660

[brb32288-bib-0051] Klingberg, T. (2010). Training and plasticity of working memory. Trends in Cognitive Sciences, 14(7), 317–324. 10.1016/j.tics.2010.05.002 20630350

[brb32288-bib-0052] Kohno, S., Miyai, I., Seiyama, A., Oda, I., Ishikawa, A., Tsuneishi, S., Amita, T., & Shimizu, K. (2007). Removal of the skin blood flow artifact in functional near‐infrared spectroscopic imaging data through independent component analysis. Journal of Biomedical Optics, 12(6), 062111. 10.1117/1.2814249 18163814

[brb32288-bib-0053] Komuro, H. (2016). Shogakusha no susokukanmeisou ni okeru shisei no kouka [The effect of posture on Susokukan meditation in beginners]. Komazawa Annual Reports of Psychology, 18, 17–25.

[brb32288-bib-0054] Koo, B., Lee, H. G., Nam, Y., Kang, H., Koh, C. S., Shin, H. C., & Choi, S. (2015). A hybrid NIRS‐EEG system for self‐paced brain computer interface with online motor imagery. Journal of Neuroscience Methods, 244, 26–32. 10.1016/j.jneumeth.2014.04.016 24797225

[brb32288-bib-0055] Kubota, Y., Sato, W., Toichi, M., Murai, T., Okada, T., Hayashi, A., & Sengoku, A. (2001). Frontal midline theta rhythm is correlated with cardiac autonomic activities during the performance of an attention demanding meditation procedure. Cognitive Brain Research, 11(2), 281–287. 10.1016/S0926-6410(00)00086-0 11275489

[brb32288-bib-0056] Lancaster, J. L., Woldorff, M. G., Parsons, L. M., Liotti, M., Freitas, C. S., Rainey, L., Kochunov, P. V., Nickerson, D., Mikiten, S. A., & Fox, P. T. (2000). Automated Tailairach atlas labels for functional brain mapping. Human Brain Mapping, 10(3), 120–131. 10.1002/1097-0193(200007)10:3<120::Aid‐Hbm30>3.0.Co;2‐8 10912591 PMC6871915

[brb32288-bib-0057] Lau, H., Rogers, R. D., & Passingham, R. E. (2006). Dissociating response selection and conflict in the medial frontal surface. Neuroimage, 29(2), 446–451. 10.1016/j.neuroimage.2005.07.050 16150611

[brb32288-bib-0058] Lippelt, D. P., Hommel, B., & Colzato, L. S. (2014). Focused attention, open monitoring and loving kindness meditation: Effects on attention, conflict monitoring, and creativity–A review. Frontiers in Psychology, 5, 1083. 10.3389/fpsyg.2014.01083 25295025 PMC4171985

[brb32288-bib-0059] Lutz, A., Slagter, H. A., Dunne, J. D., & Davidson, R. J. (2008). Attention regulation and monitoring in meditation. Trends in Cognitive Sciences, 12(4), 163–169. 10.1016/j.tics.2008.01.005 18329323 PMC2693206

[brb32288-bib-0060] Luu, K., & Hall, P. A. (2017). Examining the acute effects of hatha yoga and mindfulness meditation on executive function and mood. Mindfulness, 8(4), 873–880. 10.1007/s12671-016-0661-2

[brb32288-bib-0061] Malinowski, P. (2013). Neural mechanisms of attentional control in mindfulness meditation. Frontiers in Neuroscience, 7, 8. 10.3389/fnins.2013.00008 23382709 PMC3563089

[brb32288-bib-0062] Markowska, A. (2013). Attention processes in mindfulness: The influence of mindfulness intervention on performing stroop based tasks. Acta Neuropsychologica, 11(4), 333–344. 10.5604/17307503.1090458

[brb32288-bib-0063] Mason, M. F., Norton, M. I., Van Horn, J. D., Wegner, D. M., Grafton, S. T., & Macrae, C. N. (2007). Wandering minds: The default network and stimulus‐independent thought. Science, 315(5810), 393–395. 10.1126/science.1131295 17234951 PMC1821121

[brb32288-bib-0064] Menezes, C. B., De Paula Couto, M. C., Buratto, L. G., Erthal, F., Pereira, M. G., & Bizarro, L. (2013). The improvement of emotion and attention regulation after a 6‐week training of focused meditation: A randomized controlled trial. Evidence‐Based Complementary and Alternative Medicine, 2013, 1–11. 10.1155/2013/984678 PMC3722783 23935694

[brb32288-bib-0065] Miyake, A., Friedman, N. P., Emerson, M. J., Witzki, A. H., Howerter, A., & Wager, T. D. (2000). The unity and diversity of executive functions and their contributions to complex “frontal lobe” tasks: A latent variable analysis. Cognitive Psychology, 41(1), 49–100. 10.1006/cogp.1999.0734 10945922

[brb32288-bib-0066] Miyoshi, T., Tanioka, K., Yamamoto, S., Yadohisa, H., Hiroyasu, T., & Hiwa, S. (2020). Revealing changes in brain functional networks caused by focused‐attention meditation using Tucker3 clustering. Frontiers in Human Neuroscience, 13, 473. 10.3389/fnhum.2019.00473 32038204 PMC6990115

[brb32288-bib-0067] Mulquiney, P. G., Hoy, K. E., Daskalakis, Z. J., & Fitzgerald, P. B. (2011). Improving working memory: Exploring the effect of transcranial random noise stimulation and transcranial direct current stimulation on the dorsolateral prefrontal cortex. Clinical Neurophysiology, 122(12), 2384–2389. 10.1016/j.clinph.2011.05.009 21665534

[brb32288-bib-0068] Murata, T., Takahashi, T., Hamada, T., Omori, M., Kosaka, H., Yoshida, H., & Wada, Y. (2004). Individual trait anxiety levels characterizing the properties of Zen meditation. Neuropsychobiology, 50(2), 189–194. 10.1159/000079113 15292676

[brb32288-bib-0069] Nishimura, Y., Tanii, H., Hara, N., Inoue, K., Kaiya, H., Nishida, A., Okada, M., & Okazaki, Y. (2009). Relationship between the prefrontal function during a cognitive task and the severity of the symptoms in patients with panic disorder: A multi‐channel NIRS study. Psychiatry Research: Neuroimaging, 172(2), 168–172. 10.1016/j.pscychresns.2009.01.001 19324535

[brb32288-bib-0070] Nouchi, R., Saito, T., Nouchi, H., & Kawashima, R. (2016). Small acute benefits of 4 weeks processing speed training games on processing speed and inhibition performance and depressive mood in the healthy elderly people: Evidence from a randomized control trial. Frontiers in Aging Neuroscience, 8, 302. 10.3389/fnagi.2016.00302 28066229 PMC5179514

[brb32288-bib-0071] Nouchi, R., Taki, Y., Takeuchi, H., Hashizume, H., Nozawa, T., Kambara, T., Sekiguchi, A., Miyauchi, C. M., Kotozaki, Y., Nouchi, H., & Kawashima, R. (2013). Brain training game boosts executive functions, working memory and processing speed in the young adults: A randomized controlled trial. PLoS ONE, 8(2), 1–13. 10.1371/journal.pone.0055518 PMC3566110 23405164

[brb32288-bib-0072] Nouchi, R., Taki, Y., Takeuchi, H., Nozawa, T., Sekiguchi, A., & Kawashima, R. (2016). Reading aloud and solving simple arithmetic calculation intervention (learning therapy) improves inhibition, verbal episodic memory, focus attention and processing speed in healthy elderly people: Evidence from a randomized controlled trial. Frontiers in Human Neuroscience, 10, 217. 10.3389/fnhum.2016.00217 27242481 PMC4868921

[brb32288-bib-0073] Ohn, S. H., Park, C. I.l, Yoo, W. K., Ko, M. H., Choi, K. P., Kim, G. M., Lee, Y. T., & Kim, Y. H. (2008). Time‐dependent effect of transcranial direct current stimulation on the enhancement of working memory. Neuroreport, 19(1), 43–47. 10.1097/WNR.0b013e3282f2adfd 18281890

[brb32288-bib-0074] Osaka, M. (2002). Nou no memocho wakingu memori [Working memory: The sketchpad in the brain] (1st ed.). Shinyosha.

[brb32288-bib-0075] Osaka, M. (2006). Wakingu memori ni okeru chui no fokasu to yokusei no nounaihyougen [Brain mechanisms of focus and inhibition of attention in working memory]. Japanese Psychological Review, 49(2), 341–357. 10.24602/sjpr.49.2_341

[brb32288-bib-0076] Osaka, M., Komori, M., Morishita, M., & Osaka, N. (2007). Neural bases of focusing attention in working memory: An fMRI study based on group differences. Cognitive, Affective, & Behavioral Neuroscience, 7(2), 130–139. 10.1093/acprof:oSo/9780198570394.003.0006 17672384

[brb32288-bib-0077] Osaka, M., Nishizaki, Y., Komori, M., & Osaka, N. (2002). Effect of focus on verbal working memory: Critical role of the focus word in reading. Memory and Cognition, 30(4), 562–571. 10.3758/BF03194957 12184557

[brb32288-bib-0078] Otsuka, K., & Miyatani, M. (2007). Nihongo reading span test ni okeru tagettogo to shigekibun no kentou [Target words and sentences for Japanese version of the reading span test]. Hiroshimadaigaku Shinri‐Gaku Kenkyū, 7, 19–33. 10.4992/pacjpa.72.0_1ev095

[brb32288-bib-0079] Paoletti, P., & Ben Soussan, T. D. (2019). The sphere model of consciousness: From geometrical to neuro‐psycho‐educational perspectives. Logica Universalis, 13(3), 395–415. 10.1007/s11787-019-00226-0

[brb32288-bib-0080] Paoletti, P., & Ben‐Soussan, T. D. (2020). Reflections on inner and outer silence and consciousness without contents according to the sphere model of consciousness. Frontiers in Psychology, 11, 1807. 10.3389/fpsyg.2020.01807 32903475 PMC7435012

[brb32288-bib-0081] Park, Y. J., & Park, Y. B. (2012). Clinical utility of paced breathing as a concentration meditation practice. Complementary Therapies in Medicine, 20(6), 393–399. 10.1016/j.ctim.2012.07.008 23131369

[brb32288-bib-0082] Pintimalli, A., Di Giuseppe, T., Serantoni, G., Glicksohn, J., & Ben‐Soussan, T. D. (2020). Dynamics of the sphere model of consciousness: Silence, space, and self. Frontiers in Psychology, 11, 548813. 10.3389/fpsyg.2020.548813 33071865 PMC7530372

[brb32288-bib-0083] Raffone, A., & Srinivasan, N. (2017). Mindfulness and cognitive functions: Toward a unifying neurocognitive framework. Mindfulness, 8(1), 1–9. 10.1007/s12671-016-0654-1

[brb32288-bib-0084] Raichle, M. E., MacLeod, A. M., Snyder, A. Z., Powers, W. J., Gusnard, D. A., & Shulman, G. L. (2001). A default mode of brain function. Proceedings of the National Academy of Sciences of the United States of America, 98(2), 676–682. 10.1073/pnas.98.2.676 11209064 PMC14647

[brb32288-bib-0085] Ridderinkhof, K. R., Van Den Wildenberg, W. P. M., Segalowitz, S. J., & Carter, C. S. (2004). Neurocognitive mechanisms of cognitive control: The role of prefrontal cortex in action selection, response inhibition, performance monitoring, and reward‐based learning. Brain and Cognition, 56(2), 129–140. 10.1016/j.bandc.2004.09.016 15518930

[brb32288-bib-0086] Robert, C., Borella, E., Fagot, D., Lecerf, T., & De Ribaupierre, A. (2009). Working memory and inhibitory control across the life span: Intrusion errors in the Reading Span Test. Memory and Cognition, 37(3), 336–345. 10.3758/MC.37.3.336 19246348

[brb32288-bib-0087] Ryuichiro, Y., & Shinobu, N. (2010). Nyuminji sentakuteki chui ga nyuminkonnan ni oyobosu eikyou —Susokukan ni yoru chui no tousei wo mochiita kentou— [The influence of pre‐sleep selective attention on sleep onset insomnia —A study of attention control by the breath counting exercise—]. Koudou Igaku Kenkyu, 15(1), 22–32.

[brb32288-bib-0088] Schelstraete, M. A., & Hupet, M. (2002). Cognitive aging and inhibitory efficiency in the Daneman and Carpenter's working memory task. Experimental Aging Research, 28(3), 269–279. 10.1080/03610730290080326 12079578

[brb32288-bib-0089] Seeley, W. W., Menon, V., Schatzberg, A. F., Keller, J., Glover, G. H., Kenna, H., Reiss, A. L., & Greicius, M. D. (2007). Dissociable intrinsic connectivity networks for salience processing and executive control. Journal of Neuroscience, 27(9), 2349–2356. 10.1523/JNEUROSCI.5587-06.2007 17329432 PMC2680293

[brb32288-bib-0090] Simons, D. J., Boot, W. R., Charness, N., Gathercole, S. E., Chabris, C. F., Hambrick, D. Z., & Stine‐Morrow, E. A. L. (2016). Do “brain‐training” programs work? Psychological Science in the Public Interest, 17(3), 103–186. 10.1177/1529100616661983 27697851

[brb32288-bib-0091] Singh, A. K., Okamoto, M., Dan, H., Jurcak, V., & Dan, I. (2005). Spatial registration of multichannel multi‐subject fNIRS data to MNI space without MRI. Neuroimage, 27(4), 842–851. 10.1016/j.neuroimage.2005.05.019 15979346

[brb32288-bib-0092] Smallwood, J., & Schooler, J. W. (2006). The restless mind. Psychological Bulletin, 132(6), 946–958. 10.1037/0033-2909.132.6.946 17073528

[brb32288-bib-0093] Sreenivasan, K. K., Curtis, C. E., & D'Esposito, M. (2014). Revisiting the role of persistent neural activity during working memory. Trends in Cognitive Sciences, 18(2), 82–89. 10.1016/j.tics.2013.12.001 24439529 PMC3964018

[brb32288-bib-0094] Takahashi, T., Murata, T., Hamada, T., Omori, M., Kosaka, H., Kikuchi, M., Yoshida, H., & Wada, Y. (2005). Changes in EEG and autonomic nervous activity during meditation and their association with personality traits. International Journal of Psychophysiology, 55(2), 199–207. 10.1016/j.ijpsycho.2004.07.004 15649551

[brb32288-bib-0095] Takeuchi, H., Nagase, T., Taki, Y., Sassa, Y., Hashizume, H., Nouchi, R., & Kawashima, R. (2016). Effects of fast simple numerical calculation training on neural systems. Neural Plasticity, 2016, 1–15. 10.1155/2016/5940634 PMC4736604 26881117

[brb32288-bib-0096] Tang, Y. Y., Hölzel, B. K., & Posner, M. I. (2015). The neuroscience of mindfulness meditation. Nature Reviews. Neuroscience, 16(4), 213–225. 10.1038/nrn3916 25783612

[brb32288-bib-0097] Tang, Y. Y., & Posner, M. I. (2014). Training brain networks and states. Trends in Cognitive Sciences, 18(7), 345–350. 10.1016/j.tics.2014.04.002 24816329

[brb32288-bib-0098] Taren, A. A., Gianaros, P. J., Greco, C. M., Lindsay, E. K., Fairgrieve, A., Brown, K. W., Rosen, R. K., Ferris, J. L., Julson, E., Marsland, A. L., & Creswell, J. D. (2017). Mindfulness meditation training and executive control network resting state functional connectivity: A randomized controlled trial. Psychosomatic Medicine, 79(6), 674–683. 10.1097/PSY.0000000000000466 28323668 PMC5489372

[brb32288-bib-0099] Telles, S., Deepeshwar, S., Naveen, K. V., & Pailoor, S. (2015). Long latency auditory evoked potentials during meditation. Clinical EEG and Neuroscience, 46(4), 299–309. 10.1177/1550059414544737 25380593

[brb32288-bib-0100] Tsuchida, Y., Katayama, J., & Murohashi, H. (2008). Relation between individual differences in working memory capacity and attentional capture in visual three‐stimuli oddball tasks. Japanese Journal of Physiological Psychology and Psychophysiology, 26(3), 217–228. 10.5674/jjppp1983.26.217

[brb32288-bib-0101] Tsuchiya, K., Yamaya, N., Shimoda, K., & Tozato, F. (2017). Gunmadaigakuban Reading Span Test no sakusei to datousei [Development and validity of Gunma University Reading Span Test]. Gunma Occupational Therapy Research, 20, 17–21.

[brb32288-bib-0102] Tsunashima, H., & Yanagisawa, K. (2009). Measurement of brain function of car driver using functional near‐infrared spectroscopy (fNIRS). Computational Intelligence and Neuroscience, 2009, 1–13. 10.1155/2009/164958 PMC2703809 19584938

[brb32288-bib-0103] Vago, D. R., & Silbersweig, D. A. (2012). Self‐awareness, self‐regulation, and self‐transcendence (S‐ART): A framework for understanding the neurobiological mechanisms of mindfulness. Frontiers in Human Neuroscience, 6, 1–30. 10.3389/fnhum.2012.00296 23112770 PMC3480633

[brb32288-bib-0104] Van Den Noort, M., Bosch, P., Haverkort, M., & Hugdahl, K. (2008). A standard computerized version of the reading span test in different languages. European Journal of Psychological Assessment, 24(1), 35–42. 10.1027/1015-5759.24.1.35

[brb32288-bib-0105] Van Den Noort, M., Bosch, P., & Hugdahl, K. (2006). Foreign language proficiency and working memory capacity. European Psychologist, 11(4), 289–296. 10.1027/1016-9040.11.4.289

[brb32288-bib-0106] Vincent, J. L., Kahn, I., Snyder, A. Z., Raichle, M. E., & Buckner, R. L. (2008). Evidence for a frontoparietal control system revealed by intrinsic functional connectivity. Journal of Neurophysiology, 100(6), 3328–3342. 10.1152/jn.90355.2008 18799601 PMC2604839

[brb32288-bib-0107] Wang, M., Yu, B., Luo, C., Fogelson, N., Zhang, J., Jin, Z., & Li, L. (2020). Evaluating the causal contribution of fronto‐parietal cortices to the control of the bottom‐up and top‐down visual attention using fMRI‐guided TMS. Cortex; a Journal Devoted to the Study of the Nervous System and Behavior, 126, 200–212. 10.1016/j.cortex.2020.01.005 32088408

[brb32288-bib-0108] Waters, L., Barsky, A., Ridd, A., & Allen, K. (2014). Contemplative education: A systematic, evidence‐based review of the effect of meditation interventions in schools. Educational Psychology Review, 27(1), 103–134. 10.1007/s10648-014-9258-2

[brb32288-bib-0109] Wenk‐Sormaz, H. (2005). Meditation can reduce habitual responding. Alternative Therapies in Health and Medicine, 11(2), 42–59. 15819448

[brb32288-bib-0110] Yasumura, A., Inagaki, M., & Hiraki, K. (2014). Relationship between neural activity and executive function: An NIRS study. ISRN Neuroscience, 2014, 1–5. 10.1155/2014/734952 PMC4045560 24967317

[brb32288-bib-0111] Ye, J. C., Tak, S., Jang, K. E., Jung, J., & Jang, J. (2009). NIRS‐SPM: Statistical parametric mapping for near‐infrared spectroscopy. Neuroimage, 44(2), 428–447. 10.1016/j.neuroimage.2008.08.036 18848897

